# An Energy-Efficient Transmission Scheme for Real-Time Data in Wireless Sensor Networks

**DOI:** 10.3390/s150511628

**Published:** 2015-05-20

**Authors:** Jin-Woo Kim, José Ramón Ramos Barrado, Dong-Keun Jeon

**Affiliations:** 1 Research Institute of Information Science and Engineering, Mokpo National University, Mokpo City 530-729, Korea; E-Mail: jjin300@gmail.com; 2 Department of Applied Physics I, University of Malaga, Avenida Cervantes, 2, Málaga 29071, Spain; E-Mail: jdk0076@hanmail.net; 3 Department of Mechatronics, Incheon National University, Incheon City, 406-772, Korea

**Keywords:** Internet of Things, IEEE 802.15.4, D2D communication, glass fiber, carbon fiber, wireless sensor network

## Abstract

The Internet of things (IoT) is a novel paradigm where all things or objects in daily life can communicate with other devices and provide services over the Internet. Things or objects need identifying, sensing, networking and processing capabilities to make the IoT paradigm a reality. The IEEE 802.15.4 standard is one of the main communication protocols proposed for the IoT. The IEEE 802.15.4 standard provides the guaranteed time slot (GTS) mechanism that supports the quality of service (QoS) for the real-time data transmission. In spite of some QoS features in IEEE 802.15.4 standard, the problem of end-to-end delay still remains. In order to solve this problem, we propose a cooperative medium access scheme (MAC) protocol for real-time data transmission. We also evaluate the performance of the proposed scheme through simulation. The simulation results demonstrate that the proposed scheme can improve the network performance.

## 1. Introduction

The Internet of things (IoT) is a novel paradigm for the further expansion of ubiquitous computing in the scenario of modern wireless communications [1]. According to the IoT paradigm, physical objects such as radio-frequency identification (RFID) tags, sensors, actuators, and mobile phones are connected to the Internet to share information about themselves and their surrounding environments. To provide Internet connectivity and mobility to things, it is necessary to use wireless technology [2]. In 1999, Kevin Ashton from the MIT Auto-ID Labs first proposed the concept of the IoT, which investigated how to bind RFID information and the Internet and to realize object localization and state recognition using wireless sensor network (WSN) and RFID technologies [3]. 

With the RFID technology, WSNs also play a crucial role in the IoT. Especially, they can be integrated with RFID systems to monitor better the information of things, *i.e.*, their location, temperature, movements, *etc.* WSNs have been used in several applications, such as environmental monitoring, e-health, intelligent transportation systems, military, and industrial plant monitoring. Also, WSNs are employed to perform strain measurements or structural health monitoring of infrastructures. Today, the function-integration in physical things plays a key role in designing competitive products and in developing WSNs. Kunadt *et al.* in [4], describe that WSNs are suitable for integration into textile-reinforced glass fiber/carbon fiber. Such WSNs enable a structure-integrated measurement and an evaluation of mechanically-induced strains. Today, most commercial WSN solutions are based on the IEEE 802.15.4 standard, which defines the physical (PHY) and medium access control (MAC) layers for low-power, low rate communications in wireless personal area networks (WPANs) [5]. 

IEEE 802.15.4 defines two types of network topologies: star topology and peer-to-peer topology. In the star topology, all data transmitted to any destination have to pass through the coordinator. Thus, a personal area network (PAN) coordinator manages the whole network and allocates the resources for communications to devices in the PAN. Devices in the network use the carrier sensing medium access/collision avoidance (CSMA/CA) algorithm as a medium access control algorithm for data transmission. The IEEE 802.15.4 standard provides the guaranteed time slot (GTS) mechanism that enables one to reserve some time slots. Because IEEE 802.15.4 devices can send real-time data without contention using the GTS mechanism, they can reduce delays by the contention for medium access and guarantee a quality of service (QoS) for the real-time data. One of the weak points of the GTS scheme is that IEEE 802.15.4 devices cannot transmit the real-time data to the destination device in the same superframe duration despite using the GTS scheme. Especially, because the IEEE 802.15.4 standard recommends setting a low duty cycle for energy saving, high delays for real-time data can occur. Also, if the link quality between coordinator and end device is degraded or the distance between coordinator and end device is long, the energy consumption increases due to the retransmission or the higher transmission power. 

In this paper, we propose a cooperative MAC structure for real-time data transmission and energy efficiency. The proposed scheme can solve the problem related to the GTS scheme defined in the IEEE 802.15.4 standard when the superframe structure is set to a low duty cycle. In the current standard, the data transmission is only allowed between the source device and the PAN coordinator in GTS slots. When the source node transmits data to other node belonging to the PAN, it first transmits data to the PAN coordinator, which stores it and then transmits the stored data in the next superframe. Therefore, as the duty cycle in the network is low, the delay time for the transmission between source node and destination node increases. Our proposed scheme can reduce the additional delay by a relay of PAN coordinator since it can transmit the real-time data in the same superframe duration. Thus, devices using the proposed scheme can transmit the real-time data with a short delay. Also, if the link quality between coordinator and device is lower than the link quality between source device and destination device or the distance between coordinator and device is longer than the distance between a source device and a destination device, the source device can directly transmit data frame to the destination device. Therefore, it can reduce the energy consumption by the retransmission or the higher transmission power. Also, it can reduce the energy consumption by contention for channel access.

## 2. Background of IoT 

### 2.1. Protocol Stack Architecture for IoT 

Since 2003, various IEEE and IETF standardization bodies started putting together a framework for the communication protocols of the emerging wireless systems. To create a standard for resource-constrained networks and devices, the IEEE builds further upon the IEEE 802.15.4 standard. The IEEE 802.15.4 standard defines low-data-rate, low-power, and short-range radio frequency transmissions for wireless personal area networks (WPANs). However, IEEE 802.15.4 does not include specifications for the higher layers of the protocol stack, which is necessary for the seamless integration of sensor nodes into the Internet. 

**Figure 1:**
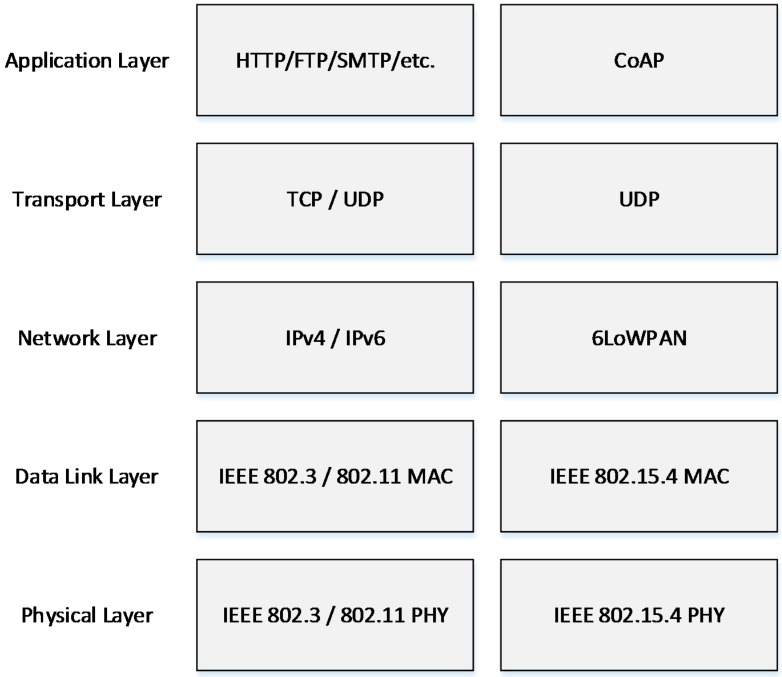
TCP/IP stack and IoT protocol stack.

In the past few years, there have been many pieces of research to enable the extension of Internet technologies to constrained devices, moving away from proprietary architectures and protocols. Most of these efforts focused on the networking layer: IPv6 over Low-Power Wireless Personal Area Networks (RFC 4919) [6], Transmission of IPv6 Packets over IEEE 802.15.4 Networks (RFC 4944) [7], IETF routing over low-power and lossy networks [8] or the ZigBee adoption of internet protocol version 6 (IPv6) [9]. These new standards enable the realization of an IoT, where end-to-end IP-based network connectivity with tiny objects such as sensors and actuators becomes possible. Based on these standardizations, a communication protocol stack for IoT is drawn in Figure 1.

### 2.2. Overview of IEEE 802.15.4

The IEEE 802.15.4 standard defines the PHY and MAC sublayer specifications for low-data-rate wireless connectivity with fixed, portable, and moving devices with no battery or very limited battery consumption requirements typically operating in a personal operating space (POS) of 10 m in size. It is foreseen that, depending on the application, a longer range at a lower data rate may be an acceptable tradeoff.

Two different device types can participate in an IEEE 802.15.4 network; a full function device (FFD) and a reduced function device (RFD). The FFD can operate in three modes serving as a PAN coordinator, a coordinator, or a device. The FFD can communicate with RFDs or other FFDs, while an RFD can only communication with the FFD. RFDs are intended for applications that are extremely simple, such as a light switch or a passive infrared sensor. They do not need to send a large amount of data and can only associate with a single FFD at a time. Consequently, the RFD can be implemented using minimal resources and memory capacity.

**Figure 2:**
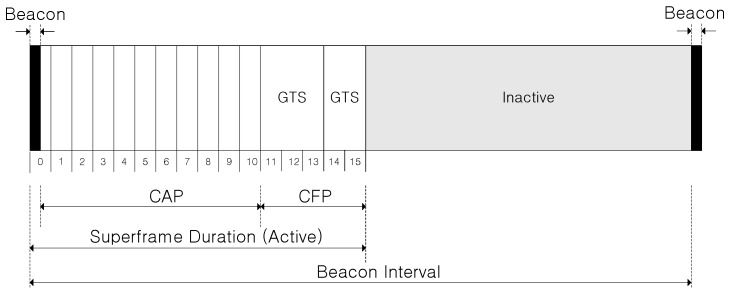
IEEE 802.15.4 superframe structure.

The IEEE 802.15.4 standard allows the optional use of a superframe structure. The superframe structure of IEEE 802.15.4 is defined by the PAN coordinator. The superframe is bounded by network beacons sent by the coordinator and is divided into 16 equally sized slots. Optionally, the superframe can have an active and an inactive portion (see Figure 2). During the inactive period, the coordinator and devices may enter a low-power mode. The beacon frame is transmitted in the first slot of each superframe. If a coordinator does not wish to use a superframe structure, it does not transmit the beacon frames. The beacons are used to synchronize the attached devices, to identify the PAN, and to describe the structure of the superframes. An IEEE 802.15.4 superframe is divided into two types of channel access period. In the contention access period (CAP), any device contends with other devices for the data transmission using a slotted CSMA/CA algorithm. For low-latency applications or applications requiring the specific data bandwidth, the PAN coordinator may dedicate portions of the active superframe to that application. These portions are called GTSs. The GTSs form the contention-free period (CFP), which always appears at the end of the active superframe starting at a slot boundary immediately following the CAP, as shown in Figure 2. The PAN coordinator may allocate up to seven of these GTSs, and a GTS may occupy more than one slot period. A sufficient portion of the CAP has to remain for contention-based access of devices in the network or new devices wishing to join the network and all contention-based transactions have to be completed before the CFP begins. Also, each device transmitting in a GTS ensures that its transaction is complete before the time of the next GTS or the end of the CFP.

To be allocated the GTS, the end device has to send a GTS request to the PAN coordinator in the CAP. When the request for the usage of GTS is accepted, the PAN coordinator advertises through its beacon that includes all the information related to the GTS allocation. The end device can use its GTS only when the beacon is received from PAN coordinator otherwise it has to wait for the next beacon. The data during the GTS first should be transmitted to PAN coordinator. Then the PAN coordinator advertises the pending address through a beacon frame so that the destination node can poll it by sending a data request command frame.

## 3. Related Works

The GTS mechanism of the IEEE 802.15.4 standard provides a reliable communication, and IEEE 802.15.4 standard allocates GTS on a first come first serve (FCFS) algorithm [10]. However, when all slots in the superframe are filled up, the other nodes in the network are denied allocation of GTS slots. This problem has created a lot of interest in the research community, and many algorithms have been proposed to overcome this problem. In [10], the authors present a more flexible approach and propose new GTS scheme for periodic real-time message allocation. Their proposed scheme divides GTS slots into more than sixteen mini time slots to enable more than seven network devices to transmit during the GTS. This scheme guarantees the delay constraints in scenarios where all GTS slots are occupied by end devices in the network. However, the proposal in [11] works under two constraints. First of all, the number of nodes requesting the GTS must be higher than seven and secondly the deadline associated with each GTS message is smaller than the beacon interval. In [12], the authors proposed an implicit GTS allocation mechanism (i-GAME), which share the GTS by several nodes in a round-robin way. In [12], based on the traffic specifications and the delay requirements, multiple devices share the same slot in multiple flows in a round robin fashion. However, iGAME cannot guarantee a GTS slot if the delay guarantee is less than the beacon interval. In [13], the authors proposed a new dynamic GTS allocation algorithm for the periodic data transmission as well as the efficient use of the GTS slots. These dynamic GTSs are allocated at regular intervals in the contention access period. The superframe back-off period unit is used to determine the length of these GTS in spite of using the superframe slot unit as defined in the standard. However, most of these algorithms cannot overcome the weak point of GTS mechanism that cannot guarantee the transmission of the real-time data in the same superframe. In [14], the authors proposed an enhanced superframe structure (ESS) algorithm to allow for a faster access to the channel and to avoid the high additional delays caused by the inactive period in the IEEE 802.15.4 standard. However, in the saturated network environment, the algorithm proposed in [14] cannot yet guarantee the transmission of the real-time data during the same superframe. Also, this algorithm has to modify the superframe structure of the IEEE 802.15.4 standard. Finally, the existing studies do not solve the network performance degradation caused by low-quality links and long distance links. 

Meanwhile, a cooperative protocol has been extensively studied in the last ten years. Especially, early research for cooperative communication mainly focused on physical layer [15,16,17,18,19,20,21]. However, the cooperative scheme is available at the different protocol layers such as MAC layer and network layer for better network performance. In particular, to facilitate access to the physical layer information and adaptation to mobility, it is natural to apply the cooperative scheme to the MAC layer [22]. In [23,24], two similar protocols, called CoopMAC and rDCF, based on the IEEE 802.11 DCF was proposed to reduce the throughput bottleneck caused by low-data-rate nodes. In [25], authors proposed an energy-efficient cooperative MAC protocol for minimizing the transmission power required for forwarding their data based on the channel state information (CSI) and relay-to-destination distance. These protocols mentioned above only extend the IEEE 802.11 standard that is not suitable for wireless sensor networks (WSN) since the physical layers of WSN devices does not support the rate adaptation scheme and consider low-power design. To reduce energy consumption, a cooperative low-power MAC (CL-MAC) protocol and automatic repeat request cooperative receiver-initiated MAC protocol (ARQ-CRI) for WSNs was proposed in [26,27]. However, because these cooperative MAC protocols do not consider cooperative communications in networks configured in a star topology, they can’t set the relay path between slave devices. In [28], authors proposed a protocol-centric approach to enable receiver cooperation and diversity combining without requiring any changes to mote hardware or the IEEE 802.15.4 LR-WPAN standard. However, this protocol only considers the peer-to-peer topology without a coordinator that manages the network. Therefore, we propose a new real-time data transmission scheme using a cooperative protocol for centralized WSNs in this paper.

## 4. Proposed Scheme

### 4.1. Basic Idea 

To overcome the weak points of GTS scheme defined by IEEE 802.15.4 standard, the following new design considerations should be applied:

- Compatible
- Low Complexity
- Reliable
- Low Power Consumption

Based on the above considerations, we propose the cooperative MAC scheme for the real-time data transmission. The proposed scheme allows one to avoid incurring in a high additional delay by storing real-time data frames in a coordinator during the inactive period. Also, the proposed scheme minimizes the energy consumption since it allows end devices to communicate directly. Our proposed scheme adds a new device-to-device (D2D) period in inactive period for real-time data transmission. 

In this paper, we assume the scenario in which the proposed scheme is applied to a local area network such as a home or an office. In WSNs, sink nodes or coordinators mainly request data from end devices or end devices periodically transmit data to sink nodes or coordinators. However, in the IoT, end devices generate data and request data from other devices. Especially, in a home network or office network, each device autonomously needs to communicate with other devices. Also, home or office networks do not require a large-scale network due to the spatial constraints. Therefore, in this paper, we assume that the proposed scheme is applied to a small-scale network such as a home network or office network. Also, in this paper, we assume that the PAN coordinator introduces an inactive period by choosing beacon order (BO) > superframe order (SO) in order to reduce energy consumption. If SO is equal to BO, we cannot use the inactive period, and the proposed scheme cannot allocate D2D slots to end devices. However, if the inactive period is removed in the superframe, the energy consumption of the devices which use IEEE 802.15.4 protocol increases greatly. Therefore, in this paper, we focus on the case that SO is smaller than BO. If SO is smaller than BO and the superframe of the IEEE 802.15.4 protocol maintains a low duty cycle, a sufficient inactive period always exists in the superframe, and the PAN coordinator can allocate D2D slots to end devices with real-time data. Thus, the proposed scheme can guarantee any QoS for D2D transmission.

The proposed scheme can reduce the end-to-end delays caused by the very low duty cycle of the IEEE 802.15.4 standard since it can transmit the real-time data to the destination device in the same superframe duration. It can also reduce the additional energy consumption caused by the transmission via the PAN coordinator since the proposed scheme can transmit the real-time data without the relay of the PAN coordinator.

**Figure 3:**
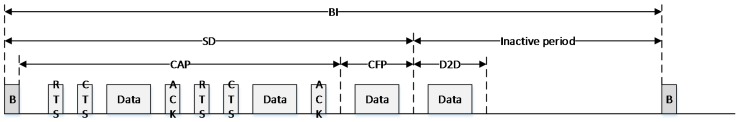
The proposed superframe structure.

The proposed scheme has the same superframe structure defined by the IEEE 802.15.4 standard. The PAN coordinator broadcasts the beacon frame at the beginning of the superframe, and it contains the information about the superframe structure. In the proposed scheme, the only period added to the superframe structure is a D2D period that is allocated for the inactive duration. Figure 3 shows the proposed superframe structure.

**Figure 4:**
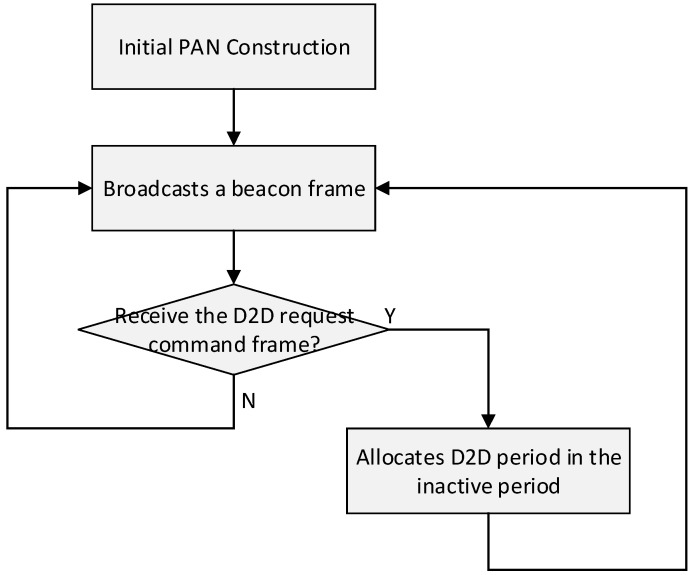
The flow chart of coordinator using the cooperative MAC structure.

As shown in Figure 3, the proposed superframe structure is based on the idea of adding a D2D period after the SD period. In the D2D period, all devices that are not related to D2D communication can go into sleep mode to save energy. In other words, devices that are not allocated a D2D slot by the PAN coordinator go into the sleep mode during the inactive period, and only devices that are allocated D2D slots transmit the data frame to the destination device in the allocated D2D period. Because the source and destination devices that are allocated D2D slot by PAN coordinator only communicate in the allocated D2D slot, they do not have to contend with other devices to transmit data frames. The key features of the proposed cooperative MAC structure include the following: firstly, in the proposed scheme, devices with real-time data do not need to contend for the channel access in the CAP since they can transmit all their data frames in the D2D period without contention. Because this feature can decrease the bandwidth and energy waste occurred by unnecessary contentions, the proposed scheme can improve the network performance. Secondly, in the proposed scheme, the source device can directly transmit the real-time data frame to the destination device in the same superframe duration. This feature allows it to avoid the additional delay occurred by storing the data on the coordinator during the inactive period and sending it out in the next superframe duration. Thirdly, our proposed scheme can exchange real-time data frames without relay by the coordinator. In the legacy GTS schemes, the PAN coordinator receives real-time data frames from the source device, transmits the received real-time data frames to the destination device, and stores real-time data frames during the inactive period. Thus, the legacy GTS schemes waste energy in coordinator relays. However, in the proposed scheme, the source device can directly transmit the real-time data frames to the destination device in the same superframe duration. Therefore, our proposed scheme can reduce the energy and resource waste by receiving real-time data frames from the source device, transmitting real-time data frames to the destination device, and storing real-time data frames during the inactive period. Lastly, our proposed superframe structure and the IEEE 802.15.4 standard have the same structure except for the addition of a D2D period. Our proposed scheme is compatible and can be directly applied with small overhead to the current IEEE 802.15.4 standard since the D2D period added in the proposed scheme is allocated to the inactive period. Figure 4 shows the flow chart of a coordinator using the cooperative MAC structure.

In Figure 4, the PAN coordinator broadcasts a beacon frame at the beginning of the superframe after it constructs PAN in the initial phase. If the PAN coordinator receives the D2D request command frame from the device with real-time data, it allocates a D2D period for devices with real-time data in the inactive period. A detailed description of each step for the proposed scheme is provided in the next subsections.

### 4.2. D2D Request Command Frame 

**Figure 5:**
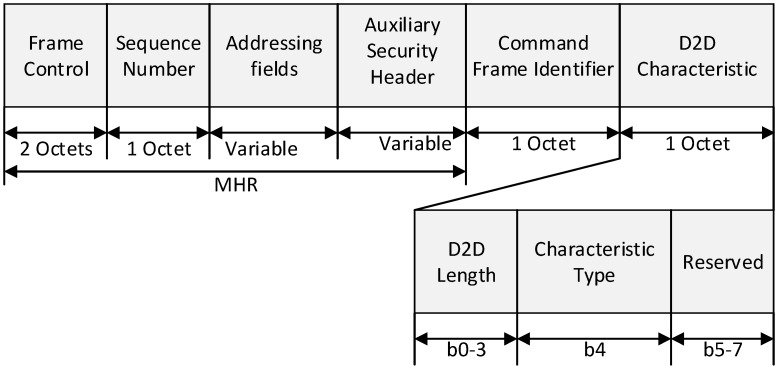
The format of a D2D request command frame.

In this subsection, we propose the D2D request command frame to support D2D communication. In the proposed scheme, devices with real-time data transmit a D2D request command frame to the coordinator for efficient channel access. The D2D request command frame is transmitted in a CAP period in the same way as other command frames defined by the IEEE 802.15.4 standard. The proposed D2D request command is used by an associated device that is requesting the allocation of a new D2D slot or the deallocation of an existing D2D slot. Also, if the RSSI or LQI value of the received frame from the coordinator is lower than the RSSI or LQI value of the destination device, the source device transmits the D2D request command frame to the coordinator. Only devices that have a short address less than 0xfffe shall send this command. The D2D request command is formatted as illustrated in Figure 5.

The MAC header (MHR) fields contain the Frame Control field, the Sequence Number field, the Addressing field, and the Auxiliary Security Header field. The Destination Addressing Mode field in Frame Control field is set to indicate the short addressing of destination device, and the Source Addressing Mode field in Frame Control field is set to indicate the short addressing of source device. The Frame Pending field in Frame Control field is set to zero and ignored upon reception, and the AR (Acknowledgment Request) field in Frame Control field is set to one. The Source PAN Identifier field in Addressing field contains the value of macPANId, and the Source Address field in Addressing field contains the value of the short address of source device. And the Destination Address field in Addressing field contains the value of the short address of destination device.

The D2D Length field contains the number of superframe slots being requested for the D2D. The Characteristics Type field is set to one if the characteristics refer to a D2D allocation or zero if the characteristics refer to a D2D deallocation.

### 4.3. Resource Management Scheme for Device-to-Device Communication 

The coordinator that receives the D2D request command frame from an end device allocates a D2D period for direct communication between end devices in the inactive period. The D2D period is allocated only by the coordinator, and it is used only for the direct communications between end devices associated with the PAN. A single time slot in D2D period may extend over one or more superframe slots. The D2D slot is allocated on the basis of the FCFS scheme, and all D2D slots are placed contiguously after the CFP period. Each D2D slot is deallocated when the direct communication between the source and the destination devices is no longer required. Also, at any time, the D2D slot can be deallocated at the discretion of the coordinator or by the device that originally requested the D2D slot. A device that has been allocated a D2D slot may also operate in the CAP. A data frame transmitted in the allocated D2D slot includes only short addressing.

The management of a D2D period is undertaken by the PAN coordinator only. To facilitate the D2D period management, the coordinator can store all the information necessary to manage the D2D period. For each D2D slot, the coordinator can store its starting slot, length, and associated device address. 

For each allocated D2D slot, the source and destination devices can store its starting slot and length. If the source device has been allocated a D2D slot, it can transmit the real-time data frame to the destination device for the entirety of the D2D slot. In the same way, the destination device can receive the real-time data from the source device for the entirety of the D2D slot. If a data frame is received during a D2D period and an acknowledgment is requested, the device transmits the acknowledgment frame as usual. If a device loses the synchronization with the coordinator, all its D2D allocations shall be lost. 

A device is instructed to request the allocation of a new D2D through the MLME-D2D.request primitive with D2D characteristics set according to the requirements of the intended application. The definition of the D2D request primitive is as follows:

MLME-D2D.request (

DeviceAddrMode,
DevicePANId,
DeviceID,
DestinationDeviceID,
D2DCharacteristics

)

To be allocated a new D2D slot, the device sends the D2D request command, as described in the previous subsection, to the coordinator. The Characteristics Type field of the D2D Characteristics field of the request is set to one (D2D allocation), and the length field is set according to the desired characteristics of the required D2D.

On receipt of the D2D request command indicating a D2D allocation request, the coordinator first checks if there is available capacity in the current superframe, based on the remaining length of the inactive period and the desired length of the requested D2D. If there is the sufficient bandwidth available, the D2D period is allocated on the basis of the FCFS scheme by the coordinator. The coordinator makes this decision within a certain superframe duration. 

**Figure 6:**
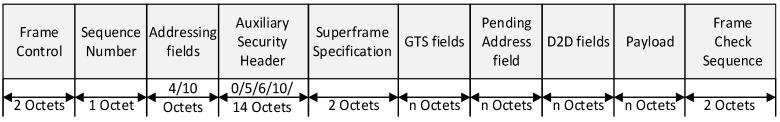
The format of the proposed beacon frame.

When the coordinator determines whether capacity is available for the requested D2D slot, it generates a D2D descriptor with the requested specifications and the short address of the requesting device. If the D2D was allocated successfully, the coordinator sets the start slot in the D2D descriptor to the superframe slot at which the D2D begins and the length in the D2D descriptor to the length of the D2D. In addition, the coordinator notifies the next higher layer of the new D2D. This notification is achieved when the MLME of the coordinator issues the MLME-D2D.indication primitive with the characteristics of the allocated D2D. If there was not sufficient capacity to allocate the requested D2D, the start slot is set to zero and the length to the largest D2D length that can currently be supported. The PAN coordinator then includes this D2D descriptor in its beacon. Figure 6 shows the format of the proposed beacon frame.

**Figure 7:**
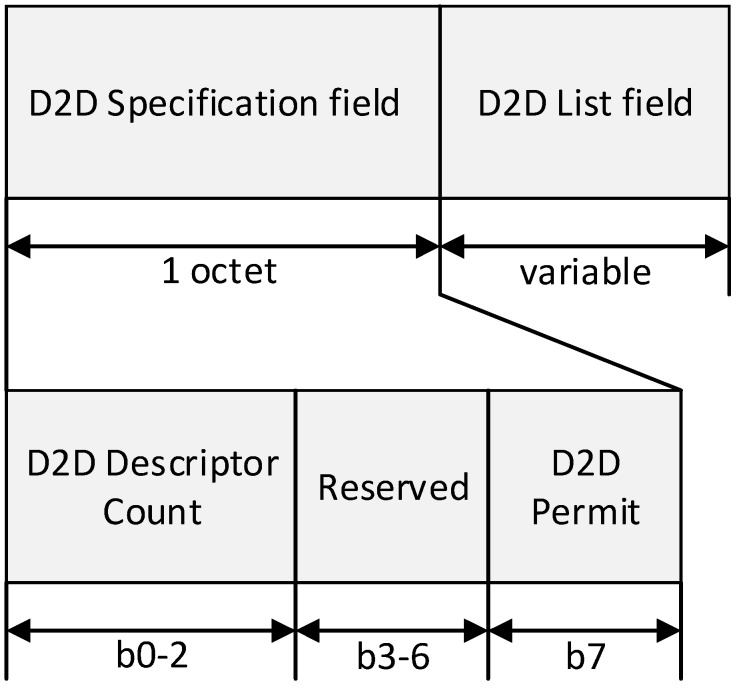
The format of the D2D field.

In Figure 6, the Frame Control field contains the information defining the frame type, addressing fields, and other control flags. The Superframe Specification field contains the information of BO, SO, CAP slot and coordinator. The GTS field contains the information of CFP period. The D2D field contains the information of D2D period and is illustrated in Figure 7.

**Figure 8:**
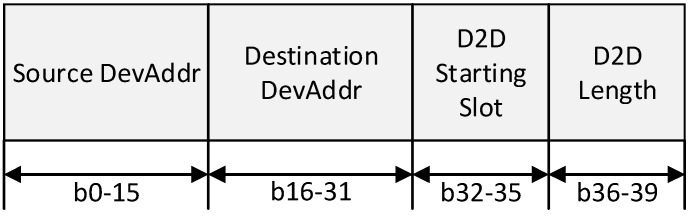
The format of D2D Descriptor.

In Figure 7, the D2D Descriptor Count field specifies the number of D2D descriptors contained in the D2D List field of the beacon frame. If the value of this field is zero, the D2D List field of the beacon frame is not present. The D2D Permit field is set to one if the coordinator is accepting D2D requests. Otherwise, the D2D Permit field shall be set to zero. The size of the D2D List field is defined by the values specified in the D2D Specification field of the beacon frame and contains the list of D2D descriptors that represents the D2D period that are being maintained. Figure 8 shows the format of each D2D Descriptor.

In Figure 8, the Source DevAddr and Destination DevAddr fields contain the short addresses of the source device and destination device for which the D2D descriptor is intended. The D2D Starting Slot field contains the superframe slot at which the D2D is to begin. The D2D Length field contains the number of contiguous superframe slots over which the D2D is active.

**Figure 9:**
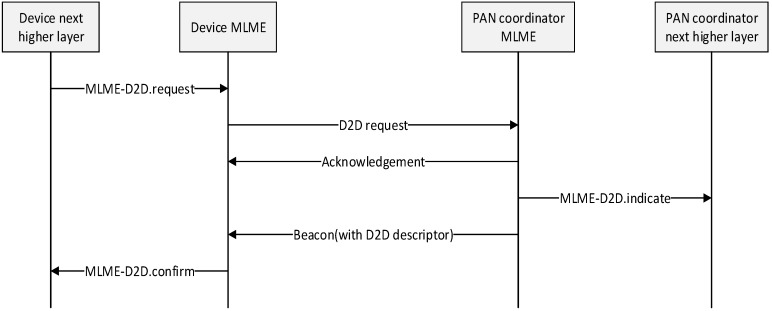
Message sequence chart for D2D allocation initiated by a device.

On receipt of a beacon frame containing a D2D descriptor corresponding to macShortAddress, the source device and destination device process the descriptor. The MLME of devices then notifies the next higher layer of whether the D2D allocation request was successful. Figure 9 shows the message flow for the case in which the device requests the D2D allocation.

Also, if device wishes to deallocate, it is instructed to request the deallocation of an existing D2D through the MLME-D2D.request primitive using the characteristics of the D2D. From this point onward, the D2D to be deallocated is not used by the device, and its stored characteristics are reset.

To request the deallocation of an existing D2D, the MLME of device transmits the D2D request command frame to the coordinator. The Characteristics Type field in the D2D request command frame is set to zero (*i.e.*, D2D deallocation), and the D2D length fields is set according to the characteristics of the D2D to deallocate. On receipt of the acknowledgment to the D2D request command, the MLME of device notifies the next higher layer of the deallocation. This notification is achieved when the MLME issues the MLME-D2D.confirm primitive with a status of SUCCESS and a D2DCharacteristics parameter with its Characteristics Type field set to zero. If the D2D request command is not received correctly by the PAN coordinator, it determines that the device has stopped using its D2D. 

On receipt of a D2D request command with the Characteristics Type field of the D2D Characteristics field set to zero (D2D deallocation), the PAN coordinator attempts to deallocate the D2D. If the D2D characteristics contained in the D2D request command frame do not match the characteristics of a known D2D, the PAN coordinator ignores the request. If the D2D characteristics contained in the D2D request command frame match the characteristics of a known D2D, the MLME of the PAN coordinator deallocates the specified D2D and notifies the next higher layer of the change. This notification is achieved when the MLME issues the MLME-D2D.indication primitive with a D2DCharacteristics parameter containing the characteristics of the deallocated D2D and a Characteristics Type field set to zero. The PAN coordinator does not add a descriptor to the beacon frame to describe the deallocation. 

D2D deallocation may be initiated by the PAN coordinator due to a deallocation request from the next higher layer or the expiration of the D2D period. The next higher layer of the PAN coordinator initiates a D2D deallocation using an MLME-D2D.request primitive with the D2D Characteristics field of the request set to indicate a D2D deallocation and the length field sets according to the characteristics of the D2D to deallocate. The MLME then responds with an MLME-D2D.confirm primitive with a status of SUCCESS and the D2DCharacteristics parameter with a Characteristics Type field set to zero.

When a D2D deallocation is initiated by the PAN coordinator either due to the D2D expiring or due to SD maintenance, the MLME of coordinator notifies the next higher layer of the change using the MLME-D2D.indication primitive with a D2DCharacteristics parameter containing the characteristics of the deallocated D2D and a Characteristics Type field set to zero.

In the case of any deallocation initiated by PAN coordinator, the PAN coordinator deallocates the D2D and add a D2D descriptor into its beacon frame corresponding to the deallocated D2D, but with its starting slot set to zero. The D2D descriptor for the deallocation remains in the beacon frame for the certain superframes duration. 

**Figure 10:**
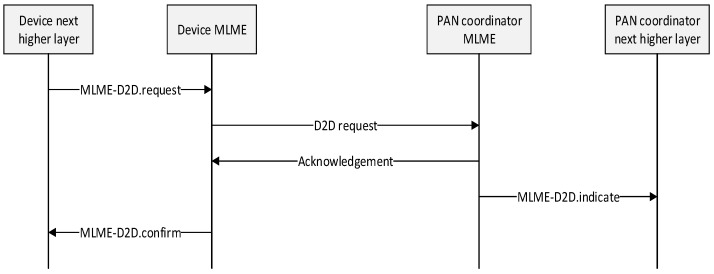
Message sequence chart for D2D deallocation initiated by a device.

On receipt of a beacon frame containing a D2D descriptor corresponding to Source DevAddr and Destination DevAddr and a start slot equal to zero, source device and destination device immediately stop using the D2D period. The MLME of devices then notifies the next higher layer of the deallocation using the MLME-D2D.indication primitive with a D2DCharacteristics parameter containing the characteristics of the deallocated D2D and a Characteristics Type field set to zero. Figure 10 shows the message flow for the cases in which a D2D deallocation is initiated by a device.

**Figure 11:**
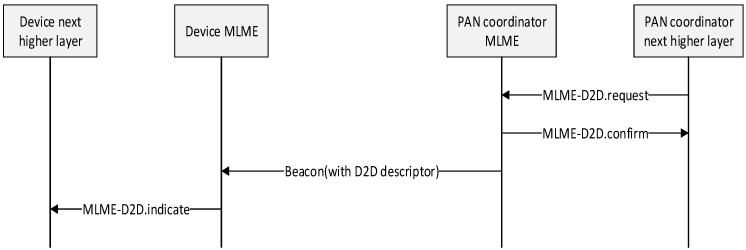
Message sequence chart for D2D deallocation initiated by a coordinator.

**Figure 12:**
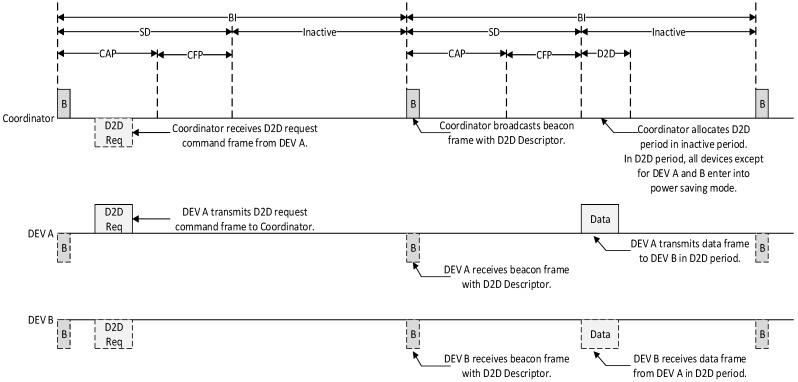
The flow of data frames and resource allocation in the proposed scheme.

Figure 11 shows the message flow for the cases in which a D2D deallocation is initiated by a coordinator. Figure 12 illustrates the flow of data frames and resource allocation in the proposed scheme.

As shown in Figure 12, the source device with the real-time data transmits the D2D request command frame in the CAP period. On receipt of the D2D request command frame, coordinator allocates a D2D period for direct communication between the source device and destination device in the inactive period. It then broadcasts a beacon frame including the information of the allocated D2D period. On receipt of a beacon frame containing the information of the assigned D2D period, source device and destination device directly exchange the real-time data without relay of coordinator. Also, to prevent unnecessary energy consumption, all devices except source device and destination device, and coordinator enter into sleep mode in all inactive periods, including the D2D period.

## 5. Performance Evaluation

We used the OMNeT++ simulator [29] to evaluate the performance of our proposed schemes, and the common simulation parameters are summarized in Table 1. The IEEE 802.15.4 model in [30] is good and was used in several papers to evaluate the performance of their proposed schemes. This model is built conforming to the latest IEEE Std. 802.15.4-2006 and implements the GTS mechanism as well as energy model. In [30], it consists of the following modules: application layer implementing the traffic generator, battery module, network module and physical layer module. The environment parameter settings are done by adjusting the variables in the omnetpp.ini configuration file of the model. The simulations are operated in beacon enabled mode, and all packets require ACK frame. The network topology used in the simulation is a star topology, where end devices directly transmit data frames to PAN coordinator. The devices are placed randomly on a plane of 50 m × 50 m size. In the simulation, to communicate with all nodes in the network, a PAN coordinator is placed in the center of the network. We used Exponential and On-OFF traffic generators for packet generation at the application module. The energy model in [31] defines four modes for the radio: Transmitting, Receiving, Idle and Sleep modes. The energy consumption is calculated by calculating the time spent on radio in each state multiplied by the energy consumption in that mode. To evaluate the consumed energy, the energy model of the CC2630, which is single chip 2.4 GHz IEEE 802.15.4-compliant RF transceiver [31] is used.

**Table 1:** Simulation Parameters.

Parameters	Value
Network topology type	Star topology
Synchronization mode	Beacon-enabled
Carrier sense sensitivity	−85 dBm
Channel number	11
IEEE 802.15.4 Header Length	22 bytes
Packet Size	50 bytes
RX current consumption	5.9 mA
TX current consumption	9.1 mA
IDLE current consumption	0.550 mA
Sleep current consumption	0.001 mA
BO	6~10
SO	5

**Figure 13:**
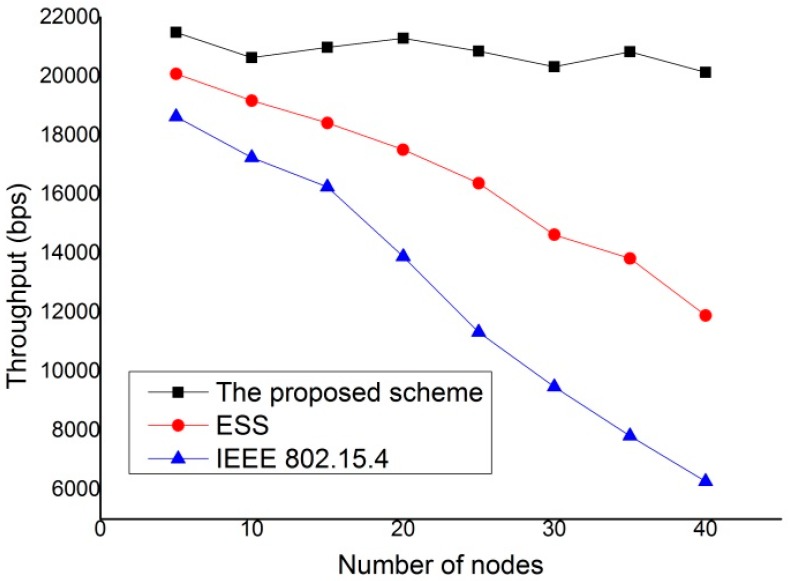
Throughput comparison.

The throughput is defined as the average number of payload bits per unit time. Therefore, it can be computed from the total delivered data by dividing by the total transmission time. Energy consumption is defined as the average energy consumed for successfully transmitting a payload from the source to the destination devices. The payload size is fixed at 50 bytes. The end-to-end delay is defined as the average delay for a single packet from source to destination devices. Figure 13 compares the throughput achieved by IEEE 802.15.4 standard, enhanced superframe structure (ESS) scheme [14] and the proposed protocol. As shown in Figure 13, the proposed scheme can improve the throughput compared with the IEEE 802.15.4 standard and ESS scheme. It can also found that the throughput of the IEEE 802.15.4 standard and ESS scheme decrease as the number of devices deployed increases. This is because more devices can contend to access medium with the increase in the number of devices. However, the throughput of the proposed scheme is not affected by the number of neighbor devices since it does not contend with neighboring devices to access medium. This feature of proposed protocol is attributed to the fact that the proposed scheme gives a steady performance regardless of number of devices in the network and is suitable for transmission of real-time traffic and isochronous traffic.

**Figure 14:**
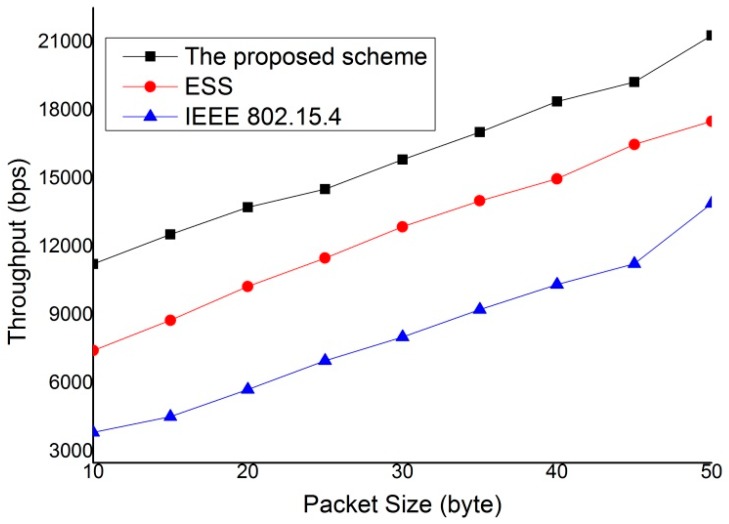
Variation of throughput with packet size given number of devices = 20.

As is well known, the size of the MAC service data unit (MSDU) has a significant impact on the efficiency of any MAC protocol. Figure 14 represents the performance of the IEEE 802.15.4 standard, ESS scheme and the proposed scheme under different data packet size. Figure 14 shows the throughput of the three schemes when the number of devices is 20. As shown in the Figure 14, the proposed scheme provides the better performance of throughput than the IEEE 802.15.4 standard and ESS scheme. As a result, the proposed scheme improves the system performance by decreasing the bandwidth waste.

**Figure 15:**
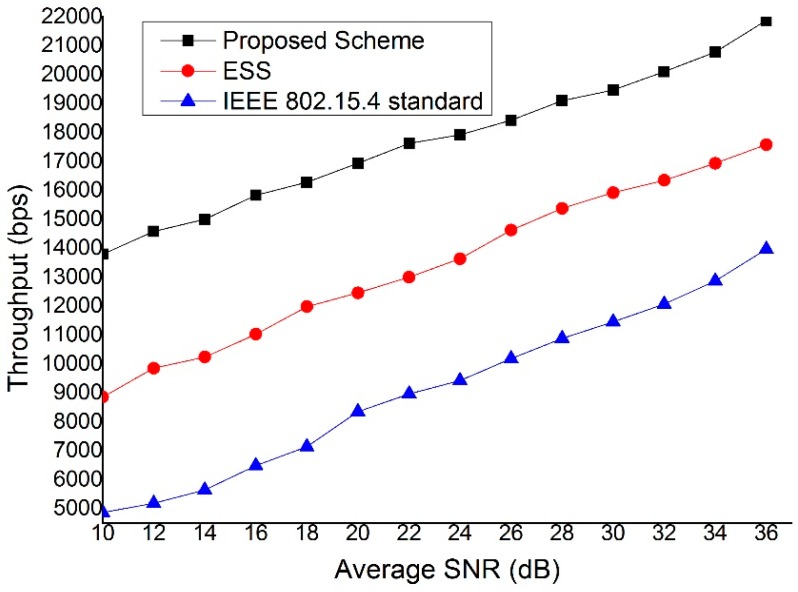
The throughput as a function of the average SNR.

Figure 15 shows the throughput as a function of the average SNR. As shown in Figure 15, the proposed scheme provides a higher throughput than both the IEEE 802.15.4 standard and ESS scheme for all SNR values. This is because the proposed scheme can select the link with the better link quality. The throughput of the ESS scheme and IEEE 802.15.4 standard is influenced more by the SNR since they have to transmit and receive data frame through the fixed link.

**Figure 16:**
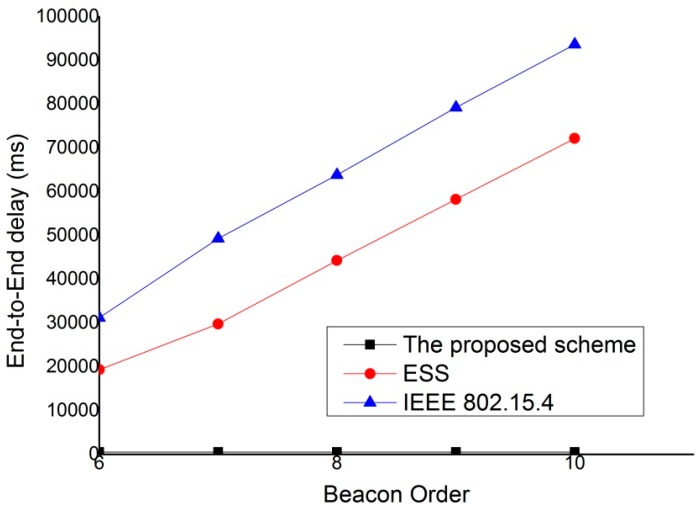
End-to-End delay according to beacon order.

Figure 16 shows the end-to-end delay according to the beacon order. SO is fixed to 5, and the network contains 40 devices. As shown in Figure 16, the proposed scheme provides a lower end to end delay than both IEEE 802.15.4 standard and ESS scheme for all BO values. This is because the proposed scheme can send and receive real-time packets without contention in the same superframe. The ESS scheme can provide the possibility to send and receive the real-time packets in the same superframe. However, as the number of devices in the network increases, the contention for CAP duration is intense. Therefore, as the value of BO increases, the end-to-end delay also increases.

**Figure 17:**
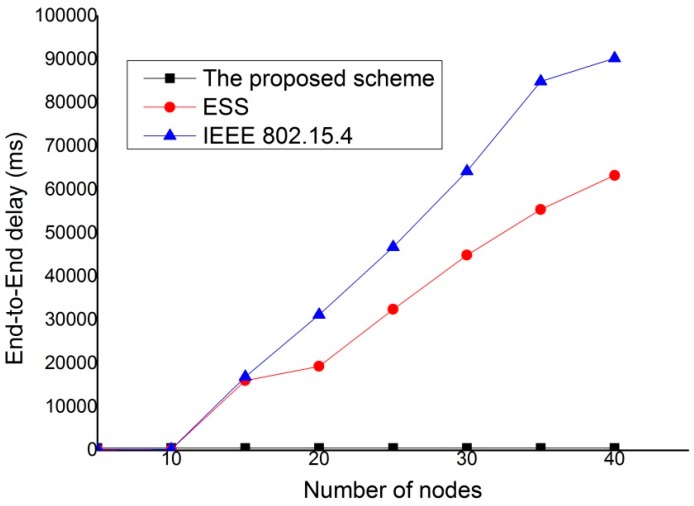
End-to-End delay for different node densities.

In Figure 17, we compare the end to end delay for different numbers of nodes. In this simulation, SO is fixed at 5 and BO is fixed to 10. The results show that the proposed scheme provides better performance than both the IEEE 802.15.4 standard and ESS scheme for all node densities. Because the proposed scheme can transmit real-time traffic without contention, it provides the constant end-to-end delay regardless of nodes densities.

**Figure 18:**
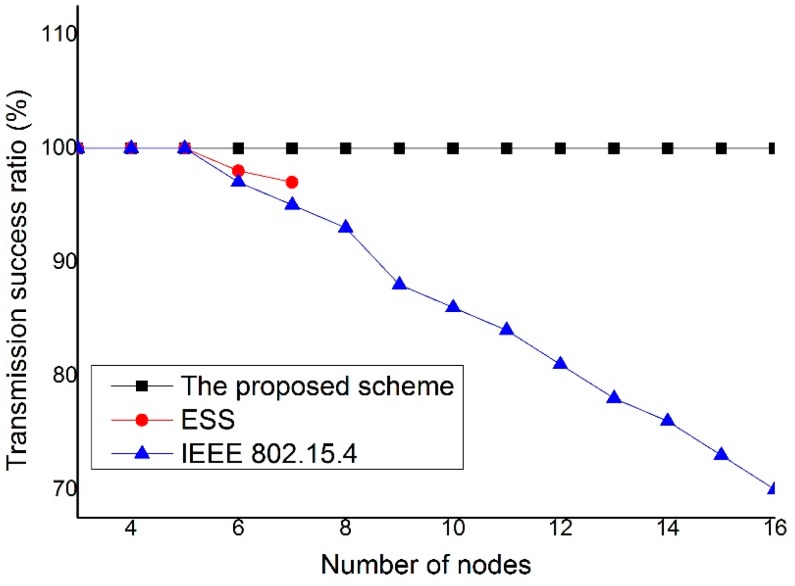
The transmission success ratio *versus* the number of nodes.

Figure 18 shows the transmission success ratio as a function of the number of nodes. As shown in Figure 18, the proposed scheme provides a higher transmission success ratio than the IEEE 802.15.4 standard and ESS scheme since it can directly transmit data frame from the source device to the destination device without contention for channel access. However, the IEEE 802.15.4 standard and ESS scheme is hard to obtain the transmission opportunity due to the contention for channel access as the number of devices increases. Notably, because the ESS scheme transmits real-time data using GTS slots, if the number of devices which use GTS slots is more than 7, the ESS scheme cannot transmit data frames using GTS slots.

**Figure 19:**
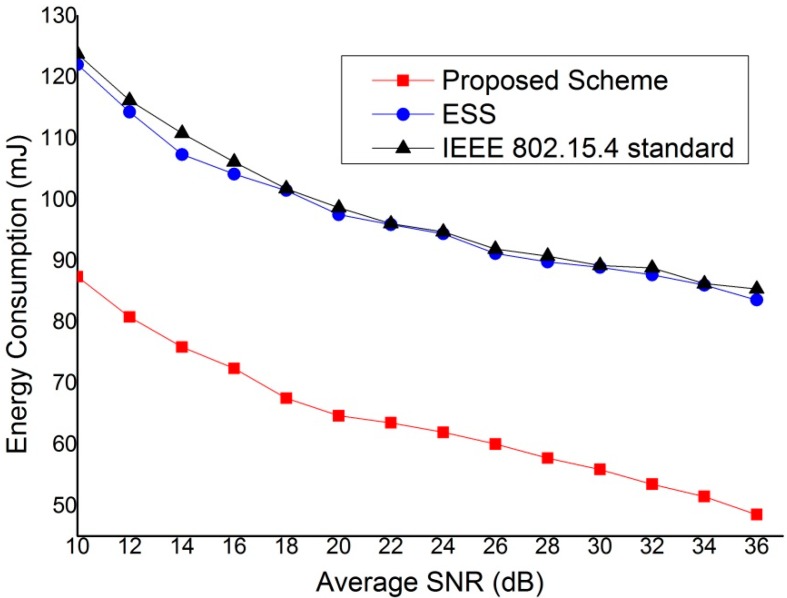
The energy consumption of device *versus* the average SNR.

In Figure 19, the dissipated power consumption of devices as a function of the average SNR is illustrated. Because the transmission failure reduces in good SNR, the expected energy consumption decreases with the average SNR. The dissipated power consumption of devices that use the proposed scheme is lower than that of devices that use the IEEE 802.15.4 standard and ESS scheme since devices adopting the proposed scheme perform the communication for real-time traffic through the path with the better link quality. However, the energy consumption of the ESS scheme and IEEE 802.15.4 standard is influenced more by the average SNR since their protocols use the links fixed via the coordinator.

**Figure 20:**
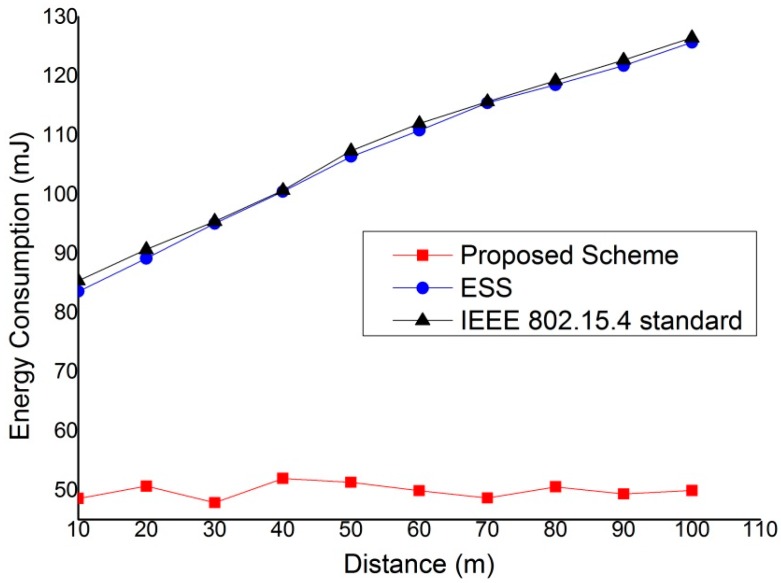
The energy consumption of device *versus* the distance between coordinator and end device.

Figure 20 shows the energy consumption of devices as a function of the distance between coordinator and the end device. In this simulation, we evaluate the energy consumption when the distance between the coordinator and end devices varies. The link quality decreases in inverse proportion to the transmission distance, and the packet error rate increases. Thus, when the distance between the source device and the destination device is long, devices have to transmit data with a higher transmission power, and the energy consumption also increases. As shown in Figure 20, the proposed scheme is not influenced by the distance between coordinator and end device since it does not use the path via coordinator and directly transmits data frames to the destination devices. Therefore, the energy consumption of the devices that use the proposed scheme does not change according to the change of distance between coordinator and the end device. However, the energy consumption of devices that use the ESS scheme and the IEEE 802.15.4 standard increases in proportion to the distance between coordinator and end device since the device has to transmit data frame using higher transmission power when the distance increases.

**Figure 21:**
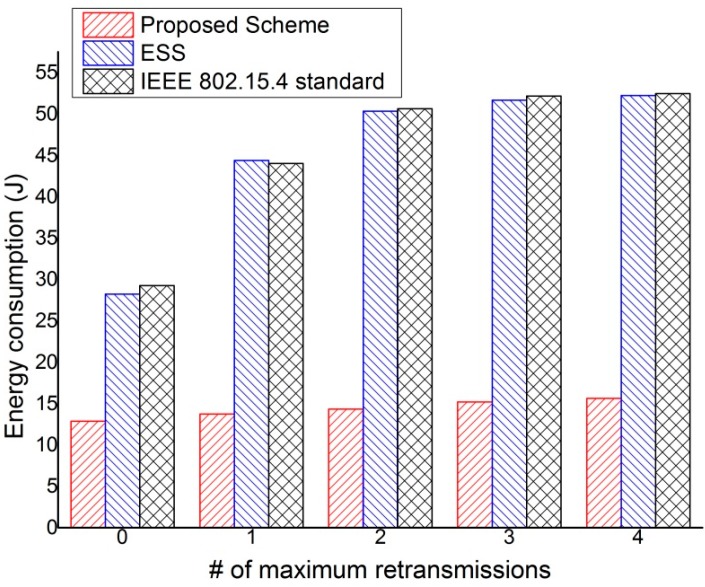
The total energy consumption of devices *versus* the number of maximum retransmissions.

Figure 21 shows the total energy consumption of all devices during a simulation run as a function of the maximum number of retransmissions. In this simulation, to compare the difference between end-to-end transmission costs with the coordinator acting as a relay and without the coordinator acting as a relay, all devices using the proposed scheme transmit the real-time data in D2D slot. Also, this simulation is operated during 20 min and the total number of devices in the network was set to 20. As shown in Figure 21, the energy consumption grows with the maximum number of retransmissions. Also, the energy consumption of the proposed scheme is smaller than the energy consumption of the ESS scheme and the IEEE 802.15.4 standard. This is because the source device can directly transmit data frames to the destination device in the proposed scheme. In both the ESS scheme and the IEEE 802.15.4 standard, the source device has to transmit data frames to the destination device through the PAN coordinator and it has to contend to send data frames with the adjacent devices. Thus, the energy of devices which use the ESS scheme and the IEEE 802.15.4 standard is consumed faster than the energy of devices which use the proposed scheme. However, in the case of the proposed scheme, because the source device can transmit data frames to the destination device without the contention, the proposed scheme can prevent the collisions caused by the contention and is not influenced by the number of maximum retransmissions. In the case of the IEEE 802.15.4 standard, it can be observed that the energy consumption of acknowledged transmissions with the maximum of four retransmissions increases 1.57 times against the energy consumption of unacknowledged transmissions. Also, it can be observed that the energy consumption of the acknowledged transmissions with the maximum of four retransmissions increases 1.85 times compared to the energy consumption of unacknowledged transmissions. In the case of the proposed scheme, it can be observed that the energy consumption of acknowledged transmission with the maximum of one retransmission increases 1.15 times against the energy consumption of unacknowledged transmissions. Also, it can be observed that the energy consumption of acknowledged transmissions with the maximum of one retransmission increases 1.21 times against the energy consumption of unacknowledged transmissions and the proposed scheme is nearly impervious to the number of retransmissions.

**Figure 22:**
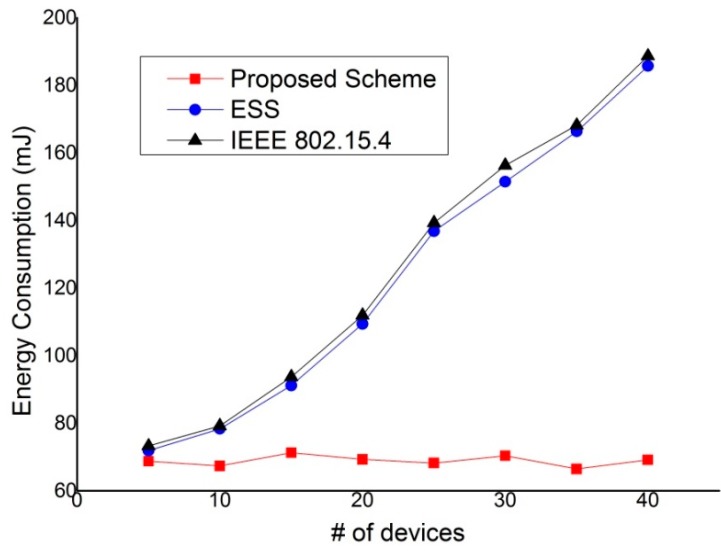
The energy consumption of device *versus* the number of devices in the network.

Figure 22 shows the energy consumption as a function of the number of nodes. In this simulation, all devices in the network except for the device with the real-time data generate and transmit data every second. Also, the device with the real-time data transmits the real-time data using the GTS mechanism of the EEE 802.15.4 standard, ESS scheme, and the proposed scheme. Then, we evaluate the energy consumption of the device that transmits the real-time data. As shown in Figure 22, the energy consumption of the device using the GTS mechanism of the IEEE 802.15.4 standard and the ESS scheme increases as the number of devices in the network increases. This is because the contention overhead for the channel access increases and the channel listening occurred by the contention or the retransmission by the collision increases as the number of nodes in the network increases. Thus, the energy consumption of the device that uses the IEEE 802.15.4 standard or the ESS scheme increases in proportion to the number of nodes. However, the energy consumption of the device that uses the proposed scheme is not influenced by the number of devices in the network since it can transmit the real-time data without contention for channel access. 

**Figure 23:**
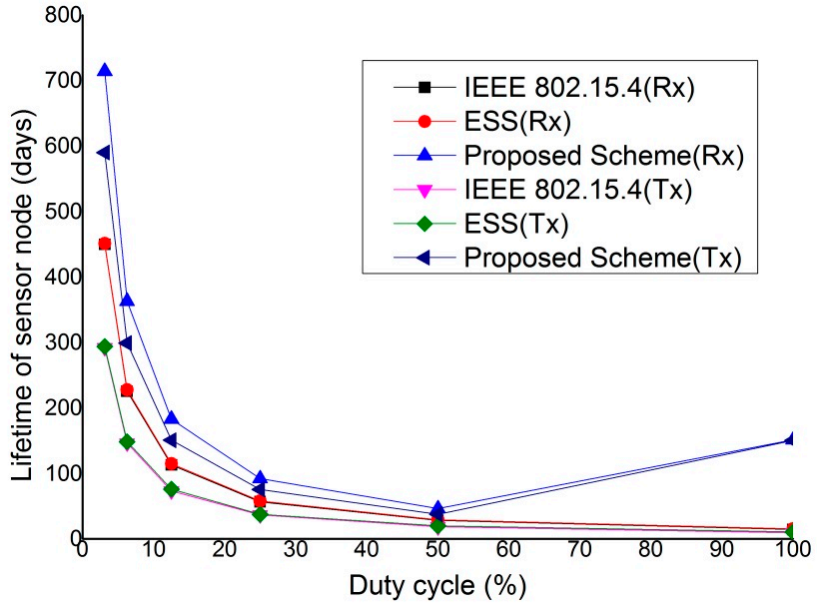
The lifetime of devices which transmit and receive the real-time data under different percentage of duty cycle.

Figure 23 shows the lifetime of a sensor node that transmits or receives the real-time data in a star topology network. To simplify the simulation, we consider the energy consumed by the RF transceiver in a node and do not consider the currents drawn by the other devices in a sensor node. We define the lifetime of a node as the expected lifetime assuming that the other nodes continue to work throughout the life of the node. The sensor nodes are powered by two AA batteries. These devices need a supply voltage in the range 3.8 V to 1.8 V [31]. The batteries are used in series, and we assume that both are rated at 2000 mAh down to 1.05 V. The lifetime of a node that uses 2000 mAh batteries in series can be obtained as:

$$\text{Mean Lifetime = }\frac{2000}{{\text{I}}_{\text{av}}}\text{ (h)}$$

where, I_av_ is the time average current (in mA) drawn from the battery. When the packet arrival rate is 0, the only power consumption is due to Rx current, *i.e.*, 5.9 mA. So the lifetime of the sensor node with 100% duty cycle is $\frac{\text{2000 mAh}}{5.9\text{ mA}}\text{ = 14.124 (days)}$. However, if the battery with the more capacities is used for sensor node or the sensor node operates with lower duty cycle, the lifetime of the sensor node increases. As shown in Figure 23, the lifetime of the device decreases in inverse proportion to the duty cycle and the lifetime of the device using the proposed scheme is longer than the lifetime of the devices that use the GTS scheme of IEEE 802.15.4 or the ESS scheme. This is because the proposed scheme can reduce the energy waste by the contention overhead. In Figure 23, when the duty cycle is 100%, the lifetime of the device using the proposed scheme increases. When the duty cycle is 100%, the inactive period in the superframe is removed, and the device using the proposed scheme cannot transmit the real-time data. Thus, it goes to the idle mode in the superframe duration and the lifetime of the device increases. 

## 6. Conclusions

In this paper, we propose a cooperative MAC protocol for real-time data transmission focusing on the beacon-enabled mode for the star network topology. Previous research works on the GTS mechanism of the IEEE 802.15.4 standard have focused on increasing utilization and reducing the bandwidth waste. However, we describe how the superframe of the current IEEE 802.15.4 standard has some drawbacks, especially for a very low duty cycle over a star topology. A newly proposed scheme solves the problem of GTS usage for a very low duty cycle through the direct transmission between end devices. The proposed scheme can also minimize the delays caused by PAN coordinator relays since devices using the proposed scheme can directly transmit the real-time data without going through a PAN coordinator. Because the proposed scheme can select the path with the better link quality, it can also reduce the energy consumption by retransmission, and increase the network performance. The simulation results show that the delay in our protocol is decreased considerably compared to both the IEEE 802.15.4 standard and the ESS scheme and the throughput is increased greatly compared to the IEEE 802.15.4 standard and the ESS scheme. The simulation results also show that the energy consumption of the proposed scheme is superior to both the IEEE 802.15.4 standard and the ESS scheme.
